# Supplementary material for: Differential expression of miRNAs in Vero cells after Mayaro virus infection
Source: Mem Inst Oswaldo Cruz. 2026 Mar 13;121:e250177. doi: 10.1590/0074-02760250177 (PMC12987633; doi:10.1590/0074-02760250177)
Supplement: Supplementary material [file 1678-8060-mioc-121-e250177-s1.pdf]

TABLE I

Sequence of miRNAs identified after 24 h of Mayaro virus infection in Vero cells and control group

Id	Mature sequence	Star sequence	Consensus precursor sequence
mml-mir-378a	acuggacuuggagucag	gcuacuuucuaguua	acuggacuuggagucaggaauaucugggguaaaaaucccaguucugcuacuuucuaguua
mmu-miR-214-3p	acagcaggcacagacaggc	cugcgugagcuugcccauca	cugcgugagcuugcccaucaacaagacagcaggcacagacaggc
mml-miR-20a-5p	caaagugcucauagugcagguag	ucuacuguagugugggcacuucc	caaagugcucauagugcagguaguuuuggcaugacucuacuguagugugggcacuucc
hsa-miR-19a-3p	ugugcaaaucuaugcaaaacuga	aguuuugcauaguugcacuac	aguuuugcauaguugcacuacaagaagaauguaguugugcaaaucuaugcaaaacuga
eca-mir-9103- iso1	uuaggcacuuuauagacauugga	uaauguuguuaagugcuuagc	uuaggcacuuuauagacauuggaucuuucauaugaguuaauguuguuaagugcuuagc
rno-mir-3570	acagucaacggucagugguuu	ucaccgacagcguugaauguucc	acagucaacggucagugguuuuuucaaaucccaaacucaccgacagcguugaauguucc
hsa-mir-2116	ccccaugcuaagaacuccuagc	uaggaguucuuagcaugggag	uaggaguucuuagcaugggaggaugucuuagcaugggaaaaccccccaugcuaagaacuccuagc
hsa-mir-5582	uuauaacuuuaagcgugccuag	ggcacgcuuaaaguuauaacuga	ggcacgcuuaaaguuauaacugacauaacuacaucaguuauaacuuuaagcgugccuag
mml-mir-500b	uaauccuugcuaccugggugagagu	ugcaccuaggcaaggauucugca	uaauccuugcuaccugggugagagugcuuucugaaugcagugcaccuaggcaaggauucugca
hsa-miR-370-3p	gccugcugggguggaaccuggu	caggucacgucucugcaguua	caggucacgucucugcaguuacacagcucacgagugccugcugggguggaaccuggu
mml-mir-7193	aggauguucuguaagccagaga	accuggcucagaggacuucuuu	accuggcucagaggacuucuuuucccagagaagaaaggauguucuguaagccagaga
hsa-mir-137	uauugcuuaagaauacgcguagu	acggguauucuuggguggauaaua	acggguauucuuggguggauaauacggauuacguuguuauugcuuaagaauacgcguagu
hsa-mir-892a	cacuguguccuuucugcguaga	uacuuagaaagguaccagucac	uacuuagaaagguaccagucacuuacauuacaugucacuguguccuuucugcguaga
hsa-mir-500b	aaugcacccaggcaaggauucu	aauccuuugucccugggugagagu	aauccuuugucccugggugagagugcuuucugaaugcaaugcacccaggcaaggauucu
hsa-miR-92b-3p	uauugcacucgucccggccucc	agggacgggacgcggugcagugu	agggacgggacgcggugcaguguuguucuuccccccgccaauauugcacucgucccggccucc
hsa-miR-664a-3p	uauucauuuauccccagccuaca	cuggcuagggaaaaugauugga	cuggcuagggaaaaugauuggauagaaaauguuauucuauucauuuauccccagccuaca
hsa-miR-502-3p	aaugcaccugggcaaggauuc	aauccuugcuaucugggugcuagug	aauccuugcuaucugggugcuagugcugucucaaugcaaugcaccugggcaaggauuc
cgr-miR-342-3p	ucucacacagaaaucgcacccguc	aggggugcuaucugugauugagggaca	aggggugcuaucugugauugagggacaugguuaauggaauugucucacacagaaaucgcacccguc
hsa-miR-129-5p	cuuuuugcggucugggcuugc	aagcccuuaccccaaaaagu	cuuuuugcggucugggcuugcuguuccucucaacaguagucaggaagcccuuaccccaaaaagu
hsa-miR-126-3p	ucguaccgugaguaauaaugcg	cauuauuacuuuugguacgcg	cauuauuacuuuugguacgcgcugugacacuucaaacucguaccgugaguaauaaugcg
hsa-miR-323b-3p	cccaauacacggucgaccucu	agguuguccguggugaguucgc	agguuguccguggugaguucgcauuauuuaaugaugcccaauacacggucgaccucu
hsa-miR-486-5p	uccuguacugagcugccccgag	cggggcagcucaguacaggaua	uccuguacugagcugccccgaggcccuucaugcugcccagcucggggcagcucaguacaggaua
hsa-miR-532-5p	caugccuugaguguaggaccgu	ccucccacacccaaggcuugca	caugccuugaguguaggaccguuggcaucuuaauuacccucccacacccaaggcuugca
hsa-mir-105-1	cugugguuccuguaugaagaca	ucuucaugcaggaaccacauug	cugugguuccuguaugaagacaguaguggugucuucaugcaggaaccacauug
hsa-miR-369-3p	aauaauacaugguugaucuuu	agaucgaccguguuauauucg	agaucgaccguguuauauucgcuuuauugacuucgaauaauacaugguugaucuuu
mmu-mir-542	ugugacagauugauaacugaaag	cucggggaucaucaugucacg	cucggggaucaucaugucacgagauaccacugugcacuugugacagauugauaacugaaag
hsa-miR-889-3p	uuaauaucggacaaccauugu	aauggcuguccguaguauggu	aauggcuguccguaguauggucucuauauuuaugaugauuaauaucggacaaccauugu
hsa-miR-654-3p	uaugucugcugaccaucacc	ugguggggccgcagaacaugugcu	uggugggccgcagaacaugugcugaguucgugccauaugucugcugaccaucacc
mml-let-7a-5p	ugagguaguagguuguaugguu	cuguacaaccuucuagcuuucc	ugagguaguagguuguaugguuuagaguuacacccugggaguuaacuguacaaccuucuagcuuucc
mml-mir-125b-1	ucccugagacccuaacuuguga	acggguuaggcucuugggagcu	ucccugagacccuaacuugugauguuuaccguuuaaauccacggguuaggcucuugggagcu
mmu-miR-7b-5p	uggaagacuagugauuuuguuguu	caacaaaucccagucugcc	uggaagacuagugauuuuguuguugucuuacugcgcucaacaacaaaucccagucugcc
hsa-miR-499a-3p	aacaucacugcaagucuuaac	cacagacuugcugugauguucac	cacagacuugcugugauguucacguggagaggaguuaaacaucacugcaagucuuaac
cpo-mir-874	uaagagacucacucacugcacuuc	ugugcaguugguggucuuucuucu	ugugcaguugguggucuuucuucuccuuucuuuaggacaguaagagacucacucacugcacuuc
hsa-mir-101-1	uacaguacugugauaacugaag	cgguuaucaugguaccgaugcu	cgguuaucaugguaccgaugcuguauaucugaaagguacaguacugugauaacugaag
hsa-mir-31	aggcaagaugcuggcauagcugu	ugcuaugccaacauauugccauc	aggcaagaugcuggcauagcuguugaacugggaaccugcuaugccaacauauugccauc
hsa-miR-92a-3p	uauugcacuugucccggccugu	ggguggggauuuguugcauuacu	ggguggggauuuguugcauuacuuguguucuauauaaaguauugcacuugucccggccugu
hsa-miR-582-3p	uaacugguugaacaacugaacc	uuacaguuguucaaccaguuacu	uuacaguuguucaaccaguuacuaaucuaacuaauuguaacugguugaacaacugaacc
hsa-miR-493-5p	uuguacaugguaggcuuucauu	ugaaggucuacugugugccagg	uuguacaugguaggcuuucauucauucguuugcacauucggugaaggucuacugugugccagg
hsa-miR-129-5p	cuuuuugcggucugggcuugc	aagcccuuaccccaaaaagcau	cuuuuugcggucugggcuugcuguacauaacucaauagccggaagcccuuaccccaaaaagcau
hsa-miR-1271-5p	cuuggcaccuagcaagcacuca	agugccugcugugugccgggc	cuuggcaccuagcaagcacucaguaaauacuuguugagugccugcugugugccgggc
mml-miR-374a-3p	cuuaucagauuguauuguaauu	uuauaauacaaccugauaagug	uuauaauacaaccugauaaguguuacagcacuuaucagauuguauuguaauu
hsa-miR-548h-5p	aaaaguaauugugguuuuugc	caaaaaccagaauuacuuuugc	aaaaguaauugugguuuuugccuuugaaaguaauggcaaaaaccagaauuacuuuugc
hsa-miR-379-5p	ugguagacuauggaacguagg	uauguaacaugguccacuaac	ugguagacuauggaacguaggcguuaugauuuuugaccuauguaacaugguccacuaac
hsa-miR-151a-3p	cuagacugaagcuccuugagg	ucgaggagcucacagucuagu	ucgaggagcucacagucuaguaugucucauccccuacuagacugaagcuccuugagg
cpo-mir-511	cuugucuuuugcucugcaguca	aauguguagcaaaagacagaau	cuugucuuuugcucugcagucaguaaauauuuuuuugugaauguguagcaaaagacagaau
mmu-miR-126b-5p	auuauuacucacgguacgaguu	cgcguaccaaaaguaauaauguc	auuauuacucacgguacgaguuugaagugucacagcgcguaccaaaaguaauaauguc
eca-mir-8964	ucccccuuaaauucugcaucaua	agggugcagauuuuaaggagau	agggugcagauuuuaaggagaucuuggccuuaagagugaguucccccuuaaauucugcaucaua
pmi-mir-2007	caugcagaauuuaccagcuucc	aaguugguaaguucugcuugac	aaguugguaaguucugcuugacuaaguugguaagucaugcagaauuuaccagcuucc
hsa-miR-92a-3p	uauugcacuugucccggccugu	agguugggaucgguugcaaugcu	agguugggaucgguugcaaugcuguguuucuguaugguauugcacuugucccggccugu
mmu-miR-214-3p	acagcaggcacagacaggcagu	ugccugucuacacuugcugugc	ugccugucuacacuugcugugcagaacauccgcucaccuguacagcaggcacagacaggcagu
mdo-miR-34a-5p	uggcagugucuuagcugguuguu	caaucagcaaguauacugcccua	uggcagugucuuagcugguuguugugagcaauaguaaggaagcaaucagcaaguauacugcccua
mml-let-7a-5p	ugagguaguaguuugugcuguu	cugcgcaagcuacugccuugcu	ugagguaguaguuugugcuguuggucggguugugacauugcccgcuguggagauaacugcgcaagcuacugccuugcu
hsa-miR-146a-5p	ugagaacugaauuccauggguu	ccugugaaauucaguucuuca	ugagaacugaauuccauggguugugucagugucagaccugugaaauucaguucuuca
hsa-miR-219b-5p	agauguccagccacaauucucg	agaauugcguuuggacaaucagu	agauguccagccacaauucucgguuggccgcagacucguacaagaauugcguuuggacaaucagu
hsa-mir-548x	agaaguuauugcaguuuuugc	aaaaacacaauuacuuuugcau	agaaguuauugcaguuuuugccauuaaaaguaauggaaaaaaacacaauuacuuuugcau
hsa-miR-10527-5p	aaagcaaauguugggugaacggc	augcacccuacucuugcuggua	aaagcaaauguugggugaacggcuguuuccucuuauuccagccaugcacccuacucuugcuggua
mmu-miR-376c-5p	guggauauuccuucuauguuua	aucauagaggaaaauccauguu	guggauauuccuucuauguuuacgugauuccugguuaaucauagaggaaaauccauguu
spu-mir-9	ucuuugguuaucuagcuguaug	auaaagcuagauaaccgaaagu	ucuuugguuaucuagcuguaugagugguguggagucuucauaaagcuagauaaccgaaagu
tch-mir-9-1	ucuuugguuaucuagcuguaug	auaaagcuagauaaccgaaagu	ucuuugguuaucuagcuguaugaguguauuggucuucauaaagcuagauaaccgaaagu
spu-mir-9isoR	ucuuugguuaucuagcuguaug	auaaagcuagauaaccgaaagu	ucuuugguuaucuagcuguaugagugccacagagccgucauaaagcuagauaaccgaaagu
spu-mir-9isoR	ugauuacgugggaucuagugac	acuagauuuuagguaagcacu	acuagauuuuagguaagcacuauugaaugcuuccaaagugauuacgugggaucuagugac
mml-miR-20a-5p	caaagugcuuacagugcagguagu	acugcagugaaggcacuuguagc	caaagugcuuacagugcagguagugauaugugcaucuacugcagugaaggcacuuguagc
mml-miR-890-3p	aacuguucccuuucugaguaga	uacuuggaaaggcaccaguu	uacuuggaaaggcaccaguuacuuagauuacacguaacuguucccuuucugaguaga

Id	Mature sequence	Star sequence	Consensus precursor sequence
eca-mir-99a-2	uagagcaaguggaccucaccagc	uggugaggcccacuuggucuag	uggugaggcccacuuggucuagggcucugacuugauccuagagcaaguggaccucaccagc
mml-mir-150	ucucccaacccuuguaccagug	ccugguacaggccugggggac	ucucccaacccuuguaccagugcugggcucagacccugguacaggccugggggac
pha-miR-30e	uguaaacauccucgacuggaagcu	cuuucagucggauguuugcagc	uguaaacauccucgacuggaagcugugaagccauagaugggcuuucagucggauguuugcagc
hsa-miR-376a-3p	aucauagaggaaaauccacgu	guagauuuuccuucuaugguu	guagauuuuccuucuaugguuacguguuugaugguuaaucauagaggaaaauccacgu
pha-mir-26b	uucaaguaauccaggauaggcu	ccuauucuugguuacuugcacg	uucaaguaauccaggauaggcugugcaggucccaaugggccuauucuugguuacuugcacg
pha-mir-26b	uucaaguaauccaggauaggcu	ccuauucuugauuacuuguuuc	uucaaguaauccaggauaggcuguuuccaucugugaggccuauucuugauuacuuguuuc
hsa-miR-96-5p	uuuggcacuagcacauuuuugcu	caaucaugugcagugccaauau	uuuggcacuagcacauuuuugcuugugucucuccgcucugagcaaucaugugcagugccaauau
hsa-miR-376a-3p	aucauagaggaaaauccacgu	gguagauucuccuucuaugagu	gguagauucuccuucuaugaguacauuauuuaugauuaaucauagaggaaaauccacgu
mml-mir-125b-1	ucccugagacccuuuaaccugug	acaggugagguucuugggagc	ucccugagacccuuuaaccugugaggacauccagggucacaggugagguucuugggagc
hsa-miR-34c-5p	aggcaguguaguuagcugauugc	aaucacuaaccacacggccagg	aggcaguguaguuagcugauugcugauaguaccaaucacuaaccacacggccagg
hsa-mir-921	aagcaggauucagacuacaauau	uguugucguucccccugcuca	aagcaggauucagacuacaauauagcugcuaagugcuguguugucguucccccugcuca
oga-miR-505	cgucaacacuugcugguuuccucu	gggagccaggaaguauugauguu	gggagccaggaaguauugauguuucugccaguuuagcgucaacacuugcugguuuccucu
sbi-MIR166e	caugguccauuuugcucugcuu	agcagagcaaaauggacugugc	caugguccauuuugcucugcuucugacagcugaguacuuugcaagcagagcaaaauggacugugc
pha-mir-152	ucagugcacuacagaacuuugu	aaaguucugagacacuccgacu	aaaguucugagacacuccgacucugaguaugauagaagucagugcacuacagaacuuugu
mml-mir-378a	acuggacuuggagucagaaggc	cuccugacuccagguccugugu	cuccugacuccagguccuguguguuaccucgaaauagcacuggacuuggagucagaaggc
hsa-miR-197-3p	uucaccaccuucuccacccagc	cggguagagagggcagugggagg	cggguagagagggcagugggagguaagagcucuucacccuucaccaccuucuccacccagc
hsa-miR-504-5p	agacccuggucugcacucuauc	agggagcgcagggcaggguuuc	agacccuggucugcacucuaucuguauucuuacugaagggagcgcagggcaggguuuc
mml-mir-15b	uagcagcacguaaauauuggcg	ccaguauuaacugugcugcugaa	uagcagcacguaaauauuggcguuaagauucuaaaauuaucuccaguauuaacugugcugcugaa
mml-miR-181c-5p	aacauucaacgcugucggugagu	accacugaccguugacuguacc	aacauucaacgcugucggugaguuugggauuugaaaaaccacugaccguugacuguacc
hsa-mir-4766	acaacaauugcucuuuuggaag	ucugaaagagcaguugguguuu	ucugaaagagcaguugguguuuauuuuuuacuaaacaacaauugcucuuuuggaag
eca-mir-1255b	uacggauaagcaaagaaaguggu	ccgcuuucuuugcucaucugua	uacggauaagcaaagaaagugguuugagccucaggaaaccgcuuucuuugcucaucugua
bmo-mir-3000	uguucaccaugccagccucug	agagguuggcaagcugaacaga	agagguuggcaagcugaacagaaucaccucugcagagagcccagggagcccacuguucaccaugccagccucug
hsa-mir-3666	ucacugccuggccucucucccuuc	agggagagaccaggcagcgg	ucacugccuggccucucucccuuccaagaucagagcagggagagaccaggcagcgg
mmu-miR-331-3p	gccccugggccuauccuagaac	cuagguaugguccccagggauc	cuagguaugguccccagggaucccagaucaaaccaggccccugggccuauccuagaac
mml-mir-15b	uagcagcacguaaauauuggcg	accaauauuacugugcugcuuc	uagcagcacguaaauauuggcguagugaaauaauguauuaaacaccaauauuacugugcugcuuc
pha-miR-30e	uguaaacauccuugacuggaagcu	cuuucagucggauguuuacagc	uguaaacauccuugacuggaagcuguaagguguucagaggagcuuucagucggauguuuacagc
pha-miR-30e	uguaaacauccuacacucagcu	cugggagguggauguuuacuuc	uguaaacauccuacacucagcuguaauacauggauuggcugggagguggauguuuacuuc
hsa-miR-107	agcagcauuguacagggcuauc	agcuucuuuacaguguugccuug	agcuucuuuacaguguugccuuguggcauggaguucaagcagcauuguacagggcuauc
mmu-miR-671-3p	uccgguucucagggcuccacc	aggaagcccuggaggggcuggagg	aggaagcccuggaggggcuggaggugauggauguuuuccuccgguucucagggcuccacc
mml-miR-181c-5p	aacauucaacgcugucggugagu	accaucgaccguugauuguacc	aacauucaacgcugucggugaguuuggaauuaaaaucaaaaccaucgaccguugauuguacc
mmu-mir-615	uccgagccugggucucccuuuu	gggggucccccggugcucggauc	gggggucccccggugcucggaucucgagggugcuuauuguucgguccgagccugggucucccuuuu
hsa-miR-186-5p	caaagaauucuccuuuugggcu	ccaaaggugaauuuuuuggga	caaagaauucuccuuuugggcuuucugguuuuauuuuaagcccaaaggugaauuuuuuggga
mml-mir-23a	aucacauugccagggauuaccacg	ggguuccuggcaugcugauu	ggguuccuggcaugcugauuugugacuuaagauuaaaaucacauugccagggauuaccacg
hsa-mir-548a	aaaaggauuugugguuuuugcc	caaaaaccgcaauuccuuuugc	aaaaggauuugugguuuuugccuguaaaaguaauggcaaaaaccgcaauuccuuuugc
hsa-mir-3173	cccugccuguuuucuccuuugu	gaaggaggaaacaggcaggccagg	cccugccuguuuucuccuuugugauuuuaugaaagcgaaggaggaaacaggcaggccagg
mze-mir-462	ugacaaggcugugauucugacu	ucagaaucacagccuugucacu	ugacaaggcugugauucugacuuagaucucuacagucuuggaucagaaucacagccuugucacu
hsa-miR-19a-3p	ugugcaaauccaugcaaaacuga	aguuuugcagguuugcauccagc	aguuuugcagguuugcauccagcugugugauauucugcugugcaaauccaugcaaaacuga
mml-mir-15b	uagcagcacagaaauauuggca	ccaauauuggcugugcugcucc	uagcagcacagaaauauuggcacagggaagcaagucugccaauauuggcugugcugcucc
mml-mir-541	ugguggggcacagaauccggacu	aaaggauucugcugucggucccacu	aaaggauucugcugucggucccacuccaaagugcauagaauggguggugggcacagaauccggacu
mml-let-7a-5p	ugagguaguaguuuguacaguu	cuguacaggccacugccuugcc	ugagguaguaguuuguacaguuugagggucuaugauaccacccgguacaggagauaacuguacaggccacugccuugcc
mdo-mir-34d	uggcaguggaguuagugauugu	aucagcuaaugacacugccuaca	uggcaguggaguuagugauuguaaccaccacaguacaaucagcuaaugacacugccuaca
hsa-mir-382	aaucauucacggacaacacuuu	gaaguuguucgugguggauucg	gaaguuguucgugguggauucgcuuuacuuaugacgaaucauucacggacaacacuuu
hsa-mir-5582	uuauaacuuuaagcgugccuag	ggcacgcuuaaaguuauaacuga	ggcacgcuuaaaguuauaacugacauaacuacaucaguuauaacuuuaagcgugccuag
aly-MIR848	uuaguuucgucuucuaccucug	uggcagaagauggaacuaaccu	uuaguuucgucuucuaccucuggaacuggaugaugucaguggcagaagauggaacuaaccu
pmi-mir-125	ucacgggacagaaccacaugcu	augugguucugucccggugagc	augugguucugucccggugagcucuguugucagaggcucacgggacagaaccacaugcu
mml-let-7a-5p	agagguaguagguugcauaguu	cuauacgaccugcugccuuucu	agagguaguagguugcauaguuuuagggcagggauuuugcccacaaggagguaacuauacgaccugcugccuuucu
dya-mir-975	ggacgugguacagaggugggg	ggccuccauaccacaguccccc	ggccuccauaccacaguccccaggcuguaagaggggacgugguacagaggugggg
mmu-mir-1956	cccuggcuggcaccuggacuua	caguccagggcuuagucagcggg	caguccagggcuuagucagcggggcccagaguggcuucccuggcuggcaccuggacuua
hsa-miR-411-5p	uaguagaccguauagcguacg	uauguaacacgguccacuaac	uaguagaccguauagcguacgcuuuaucugugacguauguaacacgguccacuaac
mml-miR-143-3p	ugagaugaagcacuguagcuca	ggugcagugcugcaucucugg	ggugcagugcugcaucucuggucaguugggagucugagaugaagcacuguagcuca
mmu-mir-6337	cacaaucucacucuguuacc	uggcagagugaguuugagucu	uggcagagugaguuugagucucugucucaaaagacacaaucucacucuguuacc
mml-mir-15b	uagcagcacauaaugguuugu	caggccauauugugcugccuca	uagcagcacauaaugguuuguggauuuugaaaaggugcaggccauauugugcugccuca
mml-miR-20a-5p	caaagugcuguucgugcagguag	acugcugagcuagcacuucccga	caaagugcuguucgugcagguagugugauuaaccgaccuacugcugagcuagcacuucccga
mml-miR-335-3p	uuuuucauuauugcuccugacc	ucaagagcaauaacgaaaaaug	ucaagagcaauaacgaaaaauguuugucauaaaccguuuuucauuauugcuccugacc
efu-mir-2683	ugacuggaagaaacucugcaaca	uggcagagguacccauuccauucc	uggcagagguacccauuccauucccagcuugcucaauagcuggugacuggaagaaacucugcaaca
mmu-miR-381-3p	uauacaagggcaagcucucugu	agcgagguugcccuuuguauauu	agcgagguugcccuuuguauauucgguuuauugacauggaauauacaagggcaagcucucugu
hsa-miR-146a-5p	ugagaacugaauuccauaggcugu	ugcccuauggacucaguucuggu	ugagaacugaauuccauaggcugugagcucuagcaaaugcccuauggacucaguucuggu
mmu-mir-140	accacaggguagaaccacggac	cagugguuuuacccuaugguag	cagugguuuuacccuaugguagguuacgucaugcuguucuaccacaggguagaaccacggac
mml-let-7a-5p	ugagguaggagguuguauaguu	cuauacggccuccuagcuuucc	ugagguaggagguuguauaguugaggaggacacccaaggagaucacuauacggccuccuagcuuucc
hsa-mir-942	uucucuguuuuggccaugugu	cacacggccgaaacagagaagg	uucucuguuuuggccaugugugacucacagccccucacacacggccgaaacagagaagg
hsa-miR-450a-5p	uuuugcgauguguuccuaauau	auugggaacauuuugcaugugu	uuuugcgauguguuccuaauauguacuauaaauauauugggaacauuuugcaugugu
hsa-miR-450a-5Iso	uuuugcgauguguuccuaauau	uuggggacauuuugcauucau	uuuugcgauguguuccuaauauguaauauaaauguauuggggacauuuugcauucau
mml-miR-20a-5p	uaaagugcuuauagugcagguag	acugcauuaugagcacuuaaagu	uaaagugcuuauagugcagguaguguuuaguuaucuacugcauuaugagcacuuaaagu
hsa-miR-219a-2-3p	agaauuguggcuggacaucugu	ugauuguccaaacgcaauucuugu	ugauuguccaaacgcaauucuuguacgagucugcggccaaccgagaauuguggcuggacaucugu
hsa-miR-135a-2-3p	auguagggauggaagccaugaa	uauggcuuuuuauuccuaugug	uauggcuuuuuauuccuaugugauaguaauaaagucucauguagggauggaagccaugaa

Id	Mature sequence	Star sequence	Consensus precursor sequence
gma-MIR10427b	aaggcccuagugcucucugcac	acaguugagcacacucugggccuc	acaguugagcacacucugggccucgguuguauguuuugaauucaaggcccuagugcucucugcac
mml-miR-548e	caaaaccugcaguuacuuuugc	aaaaguaauugugguuugugc	aaaaguaauugugguuugugccauaaauuuuacugcaaaaccugcaguuacuuuugc
mml-miR-412-5p	uggucgaccaguuggaaaguaau	uacuucaccugguccacuagc	uggucgaccaguuggaaaguaauuguuucuaauguacuucaccugguccacuagc
hsa-mir-485	agaggcuggccgugaugaauucg	gucauacacggcucuccucucu	agaggcuggccgugaugaauucgauucaucaaagcgagucauacacggcucuccucucu
lja-MIR11114	uuucuuuuccagaacccagagc	ucugguuugggaacagaaaga	uuucuuuuccagaacccagagcgacuguuacaugaucagcucugguuugggaacagaaaga
hsa-mir-105-1	ugaugggugaauuuguagaagg	uucuuuaaauucauccaucaua	uucuuuaaauucauccaucauauuaucuagaauggugaugggugaauuuguagaagg
hsa-miR-487a-3p	aaucauacagggacauccaguu	gugguuaucccugcuguguucg	gugguuaucccugcuguguucgcuuuauuuaugacgaaucauacagggacauccaguu
mml-miR-181c-5p	aacauucauuguugucggugggu	cccaccgggggaugaauguc	aacauucauuguugucgguggguugugaggaccgaggccagacccaccgggggaugaauguc
hsa-miR-18b-5p	uaaggugcaucuagugcaguuag	uacugcccuaaaugccccuucu	uaaggugcaucuagugcaguuagugaagcagcuuagaaucuacugcccuaaaugccccuucu
mml-miR-7202-5p	uugggugacauuuguagaugu	uuuacacgugucgcucgggacg	uugggugacauuuguagaugucucuguaucuugacauuuacacgugucgcucgggacg
mml-miR-548h-3p	caaaaacugcagugacuucugu	agaaguaauugcuguuuuugcc	agaaguaauugcuguuuuugccauuacuuuuaaagacaaaaacugcagugacuucugu
hsa-miR-1301-3p	uugcagcugccugggagugacuuc	ggucgcucuaggcaccgcagc	ggucgcucuaggcaccgcagcacugugcuggggauguugcagcugccugggagugacuuc
mml-mir-199a-2	acaguagucugcacauugguu	cccaguguuuagacuaucuguuc	cccaguguuuagacuaucuguucaggacucccaaauuguacaguagucugcacauugguu
hsa-miR-628-5p	augcugacauauuuacuagagg	ucuaguaagaguggcagucgaagg	augcugacauauuuacuagaggguaaaauuaauaaccuucuaguaagaguggcagucgaagg
tgu-mir-215	augaccuaugaauugacagaca	ucugucauuucuuuaggccaau	augaccuaugaauugacagacacuauagcugaguuugucugucauuucuuuaggccaau
mml-let-7a-5p	ugagguaguagguugugugguu	cuauacaaccuacugccuuccc	ugagguaguagguugugugguuucagggcagugauguugccccucagaagauaacuauacaaccuacugccuuccc
pha-mir-26b	uucaaguaauucaggauagguu	ccuguucuccauuacuuggcuc	uucaaguaauucaggauagguugugugcuguccagccuguucuccauuacuuggcuc
hsa-miR-191-5p	caacggaaucccaaaagcagcug	cugcgcuuggauuucgucccc	caacggaaucccaaaagcagcuguugucuccagagcauuccagcugcgcuuggauuucgucccc
hsa-mir-548x	agaaguuauugcaguuuuugc	caaaaacuacaauuacuuuugc	agaaguuauugcaguuuuugcaauacguuggcaaaaacuacaauuacuuuugc
hsa-miR-3164	ugugacuuuaagggaaaugacg	ccguuuugcuugaagucgcagu	ugugacuuuaagggaaaugacgcacagcaggcccuggaaucacgccguuuugcuugaagucgcagu
tca-mir-iab-4	cucggaauguaucuuaaguguu	acacuuaagauacauucugacuc	cucggaauguaucuuaaguguuuagugcuugacacacacuuaagauacauucugacuc
hsa-miR-106b-3p	ccgcacugugguacuugcugc	uaaagugcugacagugcagaua	uaaagugcugacagugcagauagugguccucuccgugcuaccgcacugugguacuugcugc
hsa-mir-3913-2	uccugggcuuuggcagacagcu	cuguugagcugagcccagggugc	uccugggcuuuggcagacagcucuuugccucucauacagcuguugagcugagcccagggugc
pha-miR-30e	uguaaacauccuacacucucagc	cugggagaaggcuguuuacucu	uguaaacauccuacacucucagcuguggaaaguaagaaagcugggagaaggcuguuuacucu
mml-miR-7180-5p	aguguacuuccugaggccucugg	uguggccucugggugugugacccuc	aguguacuuccugaggccucuggguaugaaauuccuguggccucugggugugugacccuc
mmu-miR-328-3p	cuggcccucucugcccuuccgu	ggggggcaggaggggcucaggg	ggggggcaggaggggcucagggagaaagugcaugcagccccuggcccucucugcccuuccgu
hsa-miR-183-5p	uauggcacugguagaauucacu	ugaauuaccgaagggccauaaa	uauggcacugguagaauucacugugaacagucucagucagugaauuaccgaagggccauaaa
hsa-miR-10b-5p	uacccuguagauccgaauuugu	caaauucguaucuaggggaau	uacccuguagauccgaauuuguguaaggaauuuuguggucacaaauucguaucuaggggaau
hsa-miR-487b-3p	aaucguacagggucauccacuu	ugguuaucccuguccuguucg	ugguuaucccuguccuguucguuuugcucaugucgaaucguacagggucauccacuu
hsa-mir-548x	gucauuuuugugaucugcagcu	uugcauagucacaaaagugauc	gucauuuuugugaucugcagcuaguauucucacuccaguugcauagucacaaaagugauc
mml-mir-410	aauauaacacagauggccugu	agguugucugugaugaguucg	agguugucugugaugaguucgcuuuuauuaaugacgaauauaacacagauggccugu
cgr-mir-210	cugugcgugugacagcggcugau	agccccugcccaccgcacacug	agccccugcccaccgcacacugcgcugccccagacccacugugcgugugacagcggcugau
sfr-mir-10499b-1	acugguacaaggguugggagaca	uguccccaggccuguaccagg	uguccccaggccuguaccagggucugagcccagcacugguacaaggguugggagaca
pha-miR-887	gugaacgggcgccaucccgaggcu	ccuugggagcccuguuagacucu	ccuugggagcccuguuagacucuggauuuuacacuuggagugaacgggcgccaucccgaggcu
hsa-miR-219a-1-3p	agaguugagucuggacgucccg	ugauuguccaaacgcaauucucg	ugauuguccaaacgcaauucucgagucuauggcucuggccgagaguugagucuggacgucccg
mmu-mir-200b	uaauacugccugguaaugaugac	caucuuacugggcagcauugga	caucuuacugggcagcauuggauggagucaggucucuaauacugccugguaaugaugac
hsa-miR-299-3p	uaugugggacgguaaaccgcu	ugguuuaccgucccacauacau	ugguuuaccgucccacauacauuuucaauauguaugugggacgguaaaccgcu
hsa-miR-365a-3p	uaaugccccuaaaaauccuuau	agggacuuuugggggcagaugug	agggacuuuugggggcagauguguuuccauuccacuaucauaaugccccuaaaaauccuuau
hsa-mir-3085	ucuggcugcuauggccccccucc	aggugccauucugagggccaggagu	aggugccauucugagggccaggaguuugauuaugugucacucuggcugcuauggccccccucc
hsa-mir-548x	agaaguuauugcaguuuuugc	aaaaacugcaauuacuuuug	agaaguuauugcaguuuuugccaugacuuuuaaugccaaaaacugcaauuacuuuug
hsa-miR-365a-3p	uaaugccccuaaaaauccuuau	agggacuuucaggggcagcugug	agggacuuucaggggcagcuguguuuucugacucagucauaaugccccuaaaaauccuuau
pha-mir-769	ugagaccucuggguucugagcu	ugggaucuccggggucuugguu	ugagaccucuggguucugagcugugauguugcucucgagcugggaucuccggggucuugguu
mml-mir-378a	ucuggacugugccuugcucugg	ugcagcauggcaccaucuugaga	ugcagcauggcaccaucuugagaucagagccaucucuggacugugccuugcucugg
hsa-miR-935	caguuaccgcuuccgcuaccgc	aguggcgggagcggccccucggc	aguggcgggagcggccccucggccauccuccgucugcccaguuaccgcuuccgcuaccgc
hsa-mir-4676	auuguuucaccacuggcucuuu	agagccaguggugagacaguga	agagccaguggugagacagugaguugauuacuucucauuguuucaccacuggcucuuu
osa-MIR169k	uuuggagguuaauuagcagcaug	uugcugccagucaccuucaaac	uuuggagguuaauuagcagcaugacuaauuuagccauugcugccagucaccuucaaac
pha-miR-30e	uguaaacaucccccgacuggaagcu	cuuucagucagauguuugcugc	uguaaacaucccccgacuggaagcuguaagacacagcuaagcuuucagucagauguuugcugc
mml-mir-605	gaaggcacugugggauuuaga	uaaaucccacggugccuucucc	uaaaucccacggugccuucuccuugggaaaaacagagaaggcacugugggauuuaga
mmu-miR-194-1	uguaacagcaacuccaugugga	ccaguggggcugcuguuaucug	uguaacagcaacuccauguggaaguguccacugauuccaguggggcugcuguuaucug
hsa-miR-598-3p	uacgucaucguugucaucguca	gcggugaucccgauggugugagc	gcggugaucccgauggugugagcuggaaaugggggugcuacgucaucguugucaucguca
mmu-miR-328-3p	uuggcccuuaucgaagcugcagc	ugaggcuuuagguggcugccuugg	ugaggcuuuagguggcugccuugggaucugaauugccccuuggcccuuaucgaagcugcagc
mml-mir-320b	aaaagcuggguugagagggugaac	gagcuuucucuucccaguucuucc	gagcuuucucuucccaguucuuccuggagucggggaaaagcuggguugagagggugaac
mml-mir-320b	aaaagcuggguugagagg	ccuucucuucccaguucuucu	ccuucucuucccaguucuucuuggagucaggaaaagcuggguugagagg
mml-mir-320b	aaaagcuggguugagagg	ucucuucccaguucuucc	ucucuucccaguucuucccagagucaggaaaagcuggguugagagg
mml-miR-122a-5p	aggaguguugcuugaggccagg	ccaacauagcaaggcucccaucu	aggaguguugcuugaggccaggaguucaagaccaaccuggccaacauagcaaggcucccaucu
hsa-miR-450b-5p	uuuugcaauauguuccugaaua	uugggaucauuuugcauccau	uuuugcaauauguuccugaauauguaguauaagcguauugggaucauuuugcauccau
mml-mir-378a	ucuggacugugccuugcucugg	auagccauugcacuucuccagcac	ucuggacugugccuugcucugggggccauagccauugcacuucuccagcac
mml-mir-29c	uagcaccauuugaaaucaguguu	cugguuucacauggugcuuagau	cugguuucacauggugcuuagauuuuuccaucuuuguaucuagcaccauuugaaaucaguguu
mmu-mir-12195	uguguacagucuuccagacca	ucuggaugacugugcacuugc	uguguacagucuuccagaccaccagagaugagugggugguucuggaugacugugcacuugc
mml-miR-423-5p	ugaggggcagagcgagacucu	agaauugcuagaacccggaagg	agaauugcuagaacccggaagguagagguugcagugagcugagaucgcaccacugcacuccagccugaggggcagagcgagacucu
hsa-miR-107	agcagcauuguacagggcuauga	ggcuucuuuacagugcugccuuguu	ggcuucuuuacagugcugccuuguugcauaggaucaagcagcauuguacagggcuauga
mml-mir-1-1	uggaauguaaagaaguauguau	acauacuucuuuauguacccaua	acauacuucuuuauguacccauaugaacauacaaugcuauggaauguaaagaaguauguau
mmu-miR-7b-5p	uggaagacuagugauuuuguuguu	caacaagucacagccggccuca	uggaagacuagugauuuuguuguucugaugugcuacgacaacaagucacagccggccuca
hsa-miR-10b-5p	uacccuguagaaccgaauuugu	acagauucgauucuaggggaau	uacccuguagaaccgaauuuguguggguauccguauagucacagauucgauucuaggggaau
mml-mir-7204	augauaacaccgaaguccgaau	uuggacuucgguguuaccauaa	uuggacuucgguguuaccauaaaggaauauauacuuaugauaacaccgaaguccgaau
mml-miR-423-5p	ugaggggcagagagcgagacuuu	agcucggucugaggccccucagu	ugaggggcagagagcgagacuuuucuauuuuccaaaagcucggucugaggccccucagu

Id	Mature sequence	Star sequence	Consensus precursor sequence
hsa-mir-155	uuaaugcuaaucgugauaggggu	cuccuacauguuagcauuaaca	uuaaugcuaaucgugauaggggguuuuuaccuccaacugacuccuacauguuagcauuaaca
mmu-miR-28a-3p	cacuagauugugagcuccugga	aaggagcucacagucuauugag	aaggagcucacagucuauugaguuuccuuucugacuuucccacuagauugugagcuccugga
hsa-mir-660	uacccauugcauaucggaguugu	accuccugugugcauggauua	uacccauugcauaucggaguuguaaauucucaaaacaccuccugugugcauggauua
mml-mir-199a-1	cccaguguucagacuaccuguuc	acaguagucugcacauugguu	cccaguguucagacuaccuguucaggaggcucucaacguguacaguagucugcacauugguu
ath-MIR854a	aagauguuucucuuccu	aagagauaaugucugugg	aagagauaaugucuguggcaucgggaaagauguuucucuuccu
mml-mir-486	uccuguacugagcugccccgag	cggggcagcucaguacaggau	uccuguacugagcugccccgagcugggcagcaugaagggccucggggcagcucaguacaggau
pal-miR-324-3p	acugccccaggugcugcuggg	cgcaucccuagggcauuggugu	cgcauccccuagggcauuggugugaaagcuggagacccacugccccaggugcugcuggg
hsa-miR-449a	uggcaguguauuguuagcuggu	ucagcuaacaugcaacugcuguc	uggcaguguauuguuagcugguugaauaugugaauggcaucagcuaacaugcaacugcuguc
hsa-miR-192-5p	cugaccuaugaauugacagcc	cugccaauuccauaggucacagg	cugaccuaugaauugacagccagugcucucgucuccccucuggcugccaauuccauaggucacagg
cja-mir-548k	caaaacuguaaugacuuuugc	aaaguaauugugguuuuugc	aaaguaauugugguuuuugccacaauuaaaauggcaaaacuguaaugacuuuugc
hsa-miR-574-3p	cacgcucaugcacacacccaca	ugagugugugugugugagugugug	ugagugugugugugugagugugugucgcuccgggguccacgcucaugcacacacccaca
mml-miR-34c-3p	aaucacuaacuccacugccauc	uaggcagugucauuagcugauu	uaggcagugucauuagcugauuguacugugguggguuacaaucacuaacuccacugccauc
hsa-mir-3925	cucuaauuuuaguucucuugcu	caggagaacuaaaaguggaguc	caggagaacuaaaaguggagucuggagaugugacaagcucuaauuuuaguucucuugcu
mml-miR-495-3p	aaacaaacauggugcacuucuu	gaaguugcccauguuauuuucg	gaaguugcccauguuauuuucgcuuuauaugugacgaaacaaacauggugcacuucuu
mml-mir-29c	uagcaccauuugaaaucaguguu	gcugguuucauaugguggguuuaga	gcugguuucauaugguggguuuagauuuaaauagugauugucuagcaccauuugaaaucaguguu
hsa-mir-555	ugcagccagcagugggaccuaagg	uagaucccagucuggcucuguu	ugcagccagcagugggaccuaagguucuagaauggccuagaucccagucuggcucuguu
hsa-mir-494	ugaaacauacacgggaaaccucu	agguuguccguguugucuucuc	agguuguccguguugucuucucuuuauuuaugaugaaacauacacgggaaaccucu
hsa-miR-301a-5p	gcucugacuuuauugcacuacu	cagugcaauaguauugucaaagcauc	gcucugacuuuauugcacuacuguacuuuacagcuagcagugcaauaguauugucaaagcauc
mmu-miR-135b-5p	uauggcuuuucauuccuauguga	auguagggcuaaaagccauggg	uauggcuuuucauuccuaugugauugcugucccaaacucauguagggcuaaaagccauggg
mml-mir-543	aaacauucgcggugcacuucuu	aaguugcccgugucuuuuucg	aaguugcccgugucuuuuucgcuuuauuugugccgaaacauucgcggugcacuucuu
hsa-miR-99b-5p	aacccguagauccgaacuugug	acaagcuugugucuauagguau	aacccguagauccgaacuugugguauuagucugcacaagcuugugucuauagguau
hsa-mir-1302-1	uccuugcuuaccagaaguuca	gaauuucagguaaacaaugaau	gaauuucagguaaacaaugaauaguuuuuuuagucuguccuugcuuaccagaaguuca
mmu-miR-132-3p	uaacagucuacagccauggucg	accguggcuuucgauuguuacu	accguggcuuucgauuguuacuguggggaacuggagguaacagucuacagccauggucg
nle-mir-582	uaacugguugaacaacuguaa	uucaguuguucaaccaguuaca	uucaguuguucaaccaguuacaauuaguuagauuaguaacugguugaacaacuguaa
mml-mir-15b	cagcagcacacugugguuugua	caaaccacacugugguguuaga	cagcagcacacugugguuuguacggcacuguggccacguccaaaccacacugugguguuaga
hsa-miR-3613-5p	uguuguacuuuuuuuuuuguuc	acaaaaaaaaaagcccaacccu	uguuguacuuuuuuuuuuguucguugcauuuuuaggaacaaaaaaaaaagcccaacccu
hsa-miR-615-5p	gggguccggugcggag	cccaccccgcccccua	cccaccccgcccccuagccagcaacggcagcgugccggggguccggugcggag
mml-mir-130a	uagugcaauauugcuuauagggu	acccuaucaauauugucucugc	acccuaucaauauugucucugcuguguaaauaguucugaguagugcaauauugcuuauagggu
mmu-miR-194-1	uguaacagcaacuccaugugga	caguggagaugcuguuacuuu	uguaacagcaacuccauguggacuguguaccaauuuccaguggagaugcuguuacuuu
hsa-miR-19a-3p	ugugcaaauccaugcaaaacuga	aguuuugcagguuugcauuuc	aguuuugcagguuugcauuucagcguauauauguauauguggcugugcaaauccaugcaaaacuga
hsa-miR-146a-5p	ugagaacugcaauuacuuuggc	aaaaguaauugugguguuuucc	aaaaguaauugugguguuuuccauuaaaaguaaugaugagaacugcaauuacuuuggc
mml-miR-154-5p	uagguuauccguguugccuucg	aaucauacacgguugaccuauu	uagguuauccguguugccuucgcuuuauuugugacgaaucauacacgguugaccuauu
hsa-mir-892b	cacuggcuccuuucugguaga	uacucagaaaggugccauuuau	uacucagaaaggugccauuuauguagauuauaugucacuggcuccuuucugguaga
mml-mir-1-1	uggaauguaaagaaguauguau	acauacuucuuuauaugcccaua	acauacuucuuuauaugcccauauggaccugcuaagcuauggaauguaaagaaguauguau
hsa-miR-218-5p	uugugcuugaucuaaccaugu	augguuccgucaagcaccaug	uugugcuugaucuaaccaugugguugcgagguaugaguaaaacaugguuccgucaagcaccaug
mml-miR-122a-5p	uggagugugacaaugguguuu	acgccauuaucacacuaaaua	uggagugugacaaugguguuugugucuaaacuaucaaacgccauuaucacacuaaaua
pha-mir-145	guccaguuuucccaggaaucccu	auuccuggaaauacuguucuu	guccaguuuucccaggaaucccuuaaaugcuaagaugggggauuccuggaaauacuguucuu
hsa-miR-409-5p	agguuacccgagcaacuuugcauc	gaauguugcucggugaaccccu	agguuacccgagcaacuuugcaucuggacgacgaauguugcucggugaaccccu
mml-miR-676-3p	ccguccuaagguuguugaguug	acucuucaaccucaggacuugca	acucuucaaccucaggacuugcagaauuaauggaaugccguccuaagguuguugaguug
mml-mir-23a	aucacauugccagggauuucc	gggguuccuggggaugggauuu	gggguuccuggggaugggauuugcuuccugucacaaaucacauugccagggauuucc
mml-mir-1-1	aggaaugugaguagccuccaga	cagaggccacucacauucuuug	cagaggccacucacauucuuuguguugagucaaggaaugugaguagccuccaga
mml-miR-423-5p	ugaggggccucagaccgagcuuu	ucucgcucucugccccucagc	ugaggggccucagaccgagcuuuuggaaaauagaaaagucucgcucucugccccucagc
mml-mir-4803	uaacauaauaguguggacuga	aaucuacacuauuaugguaaa	uaacauaauaguguggacugaaucacacacacauuucaaucuacacuauuaugguaaa
mml-mir-184	uggacggagaacugauaagggu	ccuuaucacuuuuccagcccagc	ccuuaucacuuuuccagcccagcuuuaugacuguaaguguuggacggagaacugauaagggu
mmu-miR-200a-3p	caacacuguacuggaagaugga	caucuuuaccagacaguguuagga	caucuuuaccagacaguguuaggagcuucacaauuagaccauccaacacuguacuggaagaugga
hsa-miR-218-5p	uugugcuugaucuaaccaugu	augguucugucaagcaccgcg	uugugcuugaucuaaccaugugguggaacgauggaaacggaacaugguucugucaagcaccgcg
mml-mir-320b	aaaagcuggguugagagggc	uuucucuucccaguuuuccc	uuucucuucccaguuuucccaaaguugggaaaagcuggguugagagggc
mmu-miR-135b-5p	uauggcuuuuuauuccuaugug	uauagggauuggagccguggc	uauggcuuuuuauuccuaugugauucuacugcucacucauauagggauuggagccguggc
mmu-miR-294-3p	aaagugcugcgacauuugagcgu	ccucaaauguggagcacuauucu	ccucaaauguggagcacuauucugauguccaaguggaaagugcugcgacauuugagcgu
mml-mir-130a	cagugcaaugauauugucaaagc	gcucugacgagguugcacuacu	gcucugacgagguugcacuacugugcucugagaagcagugcaaugauauugucaaagc
mml-miR-204-3p	ugcugggacgugugguuucu	agacuacaacucccagaaug	agacuacaacucccagaaugcagcggggccuaucggggauagugauugcccggaauauggcaugcugggacgugugguuucu
mmu-miR-200a-3p	uaacacugucugguaacgaug	caucuuaccggacagugcugga	caucuuaccggacagugcuggauuucccagcuugacucuaacacugucugguaacgaug
mml-mir-203	gugaaauguuuaggaccacuag	agugguucuuaacaguucaacag	agugguucuuaacaguucaacaguucuguagcgcaauugugaaauguuuaggaccacuag
mml-miR-222-3p	agcuacaucuggcuacugggucucu	ucaguagccaguguagauccug	ucaguagccaguguagauccugucuuucguaaucagcagcuacaucuggcuacugggucucu
mml-miR-499-5p	uuaagacuugcagugauguuu	acaucacagcaagucugugcu	uuaagacuugcagugauguuuaacuccucuccacgugaacaucacagcaagucugugcu
hsa-mir-4797	aguaaguggcacucugucuuc	aagacagagugccacuuacuga	aagacagagugccacuuacugagagaaaaaaccuuucaguaaguggcacucugucuuc
mml-mir-378a	ccuggacuucucauguuugcuc	aaaaaugggaaguccaaggac	ccuggacuucucauguuugcucuugagcugccugagcaaaaaugggaaguccaaggac
oan-mir-29a-1	augguaaugacugauuucuuugg	uaagaaauaggucauuaacaguag	uaagaaauaggucauuaacaguaguuaacguuuacuugugaucuaugguaaugacugauuucuuugg
hsa-mir-548ao	caaagacugugauuacauuagc	aaaaguaauugcgguuuuuacc	aaaaguaauugcgguuuuuaccauuacuuucaauggcaaagacugugauuacauuagc
hsa-miR-584-5p	uuaugguuugccugggacuga	ucaguuccaggccaaccaggcu	uuaugguuugccugggacugaggaauuugcugggauaugucaguuccaggccaaccaggcu
mml-mir-548g	agaguaauugugguuucuguc	aaaaaccacaauuacuuuugca	agaguaauugugguuucugucaugaaaagugugggcaaaaaccacaauuacuuuugca
mmu-miR-130b-5p	acucuuucccuguugcacuacu	cagugcaaugaugaaagggcau	acucuuucccuguugcacuacugugggccacugggaagcagugcaaugaugaaagggcau
hsa-miR-652-3p	aauggcgccacuaggguugugc	caacccuaggagagggugccauuc	caacccuaggagagggugccauucacauagacuauaauugaauggcgccacuaggguugugc
hsa-miR-656-3p	aauauuauacagucaaccucu	agguugccugugagguguuc	agguugccugugagguguucacuuucuauaugaugaauauuauacagucaaccucu
mml-let-7a-5p	ugagguaguagauuguauaguu	cuauacaaucuauugccuuccc	ugagguaguagauuguauaguugugggguagugauuuuacccuguucaggagauaacuauacaaucuauugccuuccc

Id	Mature sequence	Star sequence	Consensus precursor sequence
mml-miR-20a-5p	aaaagugcuuacagugcagguag	uacugcaaugcaagcacuucuu	aaaagugcuuacagugcagguagcuuuuugagaucuacugcaaugcaagcacuucuu
mml-let-7a-5p	ugagguaguagauuguauaguu	cuauacagucuacugucuuucc	ugagguaguagauuguauaguuuuagggucauaccccaucuuggagauaacuauacagucuacugucuuucc
tca-mir-11619c	uaaaaaugcagagucucaguuc	auugaaaucugcauuuuagcc	uaaaaaugcagagucucaguucucaugccagacccacugaauugaaaucugcauuuuagcc
hsa-miR-196b-5p	uagguaguuuccuguuguuggg	ucgacagcacgacacugccuuca	uagguaguuuccuguuguugggauccaccuuucucucgacagcacgacacugccuuca
mml-let-7a-5p	ugagguaguaaguuguauuguu	cuauacaacuuacuacuuuccc	ugagguaguaaguuguauuguuguggguagggauauuaggccccaauuagaagauaacuauacaacuuacuacuuuccc
bta-mir-2456	ucuggccgcgcuguccuggcaac	gccgggacggugcggcuucugc	gccgggacggugcggcuucugccguugcugcucuggccgcgcuguccuggcaac
hsa-miR-27b-3p	uucacaguggcuaaguucugc	agagcuuagccgauuggugaac	agagcuuagccgauuggugaacagugauugguuuccgcuuuguucacaguggcuaaguucugc
ptr-mir-1271	uguggcugugugcugaggguc	ucucaggacgccgcugcacgug	uguggcugugugcugagggucuguggucccugcgacucucaggacgccgcugcacgug
mml-mir-22	aagcugccaguugaagaacugu	aguucuucaguggcaagcuuu	aguucuucaguggcaagcuuuauguccugacccagcuaaagcugccaguugaagaacugu
ssa-mir-27a-2	uucacaguggcuaaguuccg	agggcuuagcugcuugugagca	agggcuuagcugcuugugagcaggguccacaccaagucguguucacaguggcuaaguuccg
hsa-miR-107	agcagcauuguacagggcuauga	agcuucuuuacagugcugccuug	agcuucuuuacagugcugccuuguagcauucaggucaagcagcauuguacagggcuauga
hsa-miR-548w	agaagucacugcaguuuuuguc	caaaaacagcaauuacuucug	agaagucacugcaguuuuugucuuuaaaaguaauggcaaaaacagcaauuacuucug
cre-MIR908	ugaggaaucggguggucaagc	cuuggccucugauuccccagc	ugaggaaucggguggucaagcagacaaggugcuuggccucugauuccccagc
mml-miR-380-3p	uauguaauaugguccacgucu	augguugaccauagaacaugcg	augguugaccauagaacaugcgcuaucucugugucguauguaauaugguccacgucu
hsa-miR-4677-3p	ucugugagaccaaagaacuacu	uuguucuuuggucuuucagc	uuguucuuuggucuuucagccaugaccugaccuucugucugugagaccaaagaacuacu
mml-mir-29c	uagcaccaucugaaaucgguu	acugauuucuuuugguguucagag	acugauuucuuuugguguucagagucaauauaauuuucuagcaccaucugaaaucgguu
eca-mir-653	auucuacuacgaucaccagagu	ccuggugaugguagcggaau	auucuacuacgaucaccagaguauaucauagcuaaaaggcccuggugaugguagcggaau
hsa-miR-374a-5p	auauaauacaaccugcuaagug	cuuagcagguuguauuaucauu	auauaauacaaccugcuaaguguccuagcacuuagcagguuguauuaucauu
hsa-miR-363-3p	aauugcacgguauccaucugua	cggguggaucacgaugcaauuuu	cggguggaucacgaugcaauuuugauuaguaucauaggagaaaaauugcacgguauccaucugua
mml-miR-181c-5p	aacauucauugcugucgguggguu	cucacugaacaaugaaugcaa	aacauucauugcugucgguggguugaacuguguagacaagcucacugaacaaugaaugcaa
pha-mir-152	ucagugcaucacagaacuuuguc	gaaguucuguuauacacucaggc	gaaguucuguuauacacucaggcuguggcucucugaaagucagugcaucacagaacuuuguc
mml-miR-181c-5p	aacauucauugcugucggugggu	cucacugaucgaugaaugcaaa	aacauucauugcugucgguggguuugagucugaaucaacucacugaucgaugaaugcaaa
hsa-mir-548an	aaaaggaauugugguuuuugac	caaaaaccgcaguuacuuuugc	aaaaggaauugugguuuuugacauuacuuuaaagucaaaaaccgcaguuacuuuugc
smo-MIR1094b	guggcuccuuguaccucuggcc	cccagaggagccuggagccacu	guggcuccuuguaccucuggccgccuguggaugggggaaggugcccagaggagccuggagccacu
mml-mir-125b-1	ucccugagacccuaacuuguga	acaagucaggcucuugggaccu	ucccugagacccuaacuugugagguauuuuaguaacaucacaagucaggcucuugggaccu
pha-mir-152	ucagugcaugacagaacuugg	agguucugugauacacuccgacu	agguucugugauacacuccgacucgggcucugcagcagucagugcaugacagaacuugg
hsa-mir-181c	accaucgaccguugaguggacc	aacauucaaccugucggugaguuu	aacauucaaccugucggugaguuugggcagcucaggcaaaccaucgaccguugaguggacc
hsa-miR-330-5p	ucucugggccugugucuuagg	caaagcacacggccugcagagagg	ucucugggccugugucuuaggcucugcaagaucaaccgagcaaagcacacggccugcagagagg
bmo-mir-2723	cagguaguccagcaaugucaga	cgcggcacuggugggccacccgc	cgcggcacuggugggccacccgcucagagccugucuugagcagguaguccagcaaugucaga
mmu-mir-7235	augggcagaagggcagcugaca	ccagcugccucuccuccaucg	ccagcugccucuccuccaucgcgcuucagaagugcugugaugggcagaagggcagcugaca
efu-mir-9193-2	ugccaggcggagggacucugg	cagguucccucuccucccagg	ugccaggcggagggacucugggcaggcccacgcuccugacccagguucccucuccucccagg
mml-mir-7192	uuguuuuuuauucugagugac	ucacucagaauuaaaaaacaau	ucacucagaauuaaaaaacaauaaauguggucugucaaggugacauuguuuuuuauucugagugac
gga-mir-219b	agaauugcguuuggacaaucagg	ggacguccagacucaacucucg	ggacguccagacucaacucucggccagagccauagacucgagaauugcguuuggacaaucagg
cin-mir-4038	ucaaggcugcauugcacugugau	agccacugcacuccagc	ucaaggcugcauugcacugugaucgcaccugugaagagccacugcacuccagc
hsa-miR-182-5p	uuuggcaaugguagaacucacacu	ugguucuagacuugccaacu	uuuggcaaugguagaacucacacuggugagguaacgggauccgguggucuagacuugccaacu
mmu-mir-496a	aguauuacauggccaaucucc	gguuguccaugguguguucauu	gguuguccauggugguguucauuuuauuuaugaugaguauuacauggccaaucucc
hsa-miR-28-5p	aaggagcuuacaaucuagcuggg	caacuagacugugagcuucua	aaggagcuuacaaucuagcugggguaaaugacuugcacaugaacacaacuagacugugagcuucua
bmo-mir-3267c	ugagccugagcucguccaacgccg	aagcuggagagcucgggccacgca	aagcuggagagcucgggccacgcagggccacaccugugagccugagcucguccaacgccg
hsa-miR-21-5p	uagcuuaucagacugauguugac	caacaccagucgaugggcuguc	uagcuuaucagacugauguugacuguugaaucucauggcaacaccagucgaugggcuguc
mml-miR-222-3p	agcuacauugucugcuggguuuc	accuggcauacaauguagauuucugu	accuggcauacaauguagauuucuguguucguuaggcaacagcuacauugucugcuggguuuc
mml-mir-1284	ucuguacagacccuggcuuuu	aaagcccauguuuguauugg	ucuguacagacccuggcuuuucuuaaauuuuauauauuggaaagcccauguuuguauugg
ath-MIR8176	cuucagagggacuuaggucuga	aggccuaagccaucucugaagaug	aggccuaagccaucucugaagaugaaaacuauccaucuucagagggacuuaggucuga
hsa-miR-340-5p	uuauaaagcaaugagacugauu	uccgucucaguuacuuuauagcc	uuauaaagcaaugagacugauugucauauguuguuuguggauccgucucaguuacuuuauagcc
hsa-miR-329-3p	aacacaccugguuaaccucuuu	gagguuuucugggguuucuguuuc	gagguuuucugggguuucuguuucuuuauugaggaugaaacacaccugguuaaccucuuu
mml-mir-616	ucucaaaacccuccaaugacu	ucauuggagaguuuugagcaga	ucucaaaacccuccaaugacuucccugacaugaaauaggaagucauuggagaguuuugagcaga
rno-mir-3549	ugggacuuuguaggccaguuga	aucucgcccgcaaagacccagc	ugggacuuuguaggccaguugauccgacacccucaucucgcccgcaaagacccagc
hsa-miR-329-3p	aacacaccugguuaaccucuuu	agagguuuucugggguuucuguuuc	agagguuuucugggguuucuguuucuuuaaugaggaugaaacacaccugguuaaccucuuu
hsa-miR-624-3p	cacaagguacugguauuacc	uaguaccaguauguuguguuca	uaguaccaguauguuguguucaguggaaccaagguaaacacaagguacugguauuacc
ssc-mir-138	agcugguguugugaaucaggccgu	cggcuacuucacaacaccagggc	agcugguguugugaaucaggccguugccaaucagagaacggcuacuucacaacaccagggc
miR-744-5p	ugcggggcuagggcuaacagc	cuguugccacuaaccucaacc	ugcggggcuagggcuaacagcagucuuacugaagguuuccuggaaaccacgcacaugcuguugccacuaaccucaacc
hsa-miR-196a-5p	uagguaguuucauguuguuggg	ucggcaacaagaaacugccugagu	uagguaguuucauguuguugggauugaguuuugaacucggcaacaagaaacugccugagu
hsa-mir-4444-1	auccccuggccccgccuccaac	aagaguuggggacaggguaccuug	aagaguuggggacaggguaccuuggcccuugugagauccccuggccccgccuccaac
ppy-mir-939	ccgugggccucugcuccccagu	cuggggagcugaggcucugggg	cuggggagcugaggcucugggggguggccggggcugaccgugggccucugcuccccagu
gga-mir-1633	augagagccugagcugcuggagc	agggcagcuuuguuucucauugg	agggcagcuuuguuucucauugggucacuuuacuggcaaugagagccugagcugcuggagc
cfa-mir-8903	auucccagccucuaucccuaga	uaggguagacccugggaau	auucccagccucuaucccuagagauucugauucaguauaucuaggguagacccugggaau
pbv-mir-132	uguggacugccaugguggccucugg	caggcucugccgcaguccacucu	uguggacugccaugguggccucuggggaaugccucccaggcccccaggcucugccgcaguccacucu
prd-lin-4	cugagauuauaggugug	augccuguaaucccaacac	cugagauuauaggugugagccaccaugccuggccuuguucucauuuuaagaaaguacagagagaggcuggacaug-guagcuuaugccuguaaucccaacac
miRNA mir-2355	uuguccuugcuguuuggagaua	auccccagauacaauggacaau	auccccagauacaauggacaauaugcuauuauaauuguauggcauuguccuugcuguuuggagaua
hsa-miR-296-3p	aggguugggguggaggcucucc	agggcccccccucaauccugu	agggcccccccucaauccuguugugccuaauucagagguugggguggaggcucucc
mml-mir-15b	uagcagcacaucaugguuuaca	cgaaucauuauuugcugcucu	uagcagcacaucaugguuuacauacuacagucaagaugcgaaucauuauuugcugcucu
csa-mir-155	ugaaaggaacacugagcugagg	cagcucaguguuccucucagauc	ugaaaggaacacugagcugagggugugcacucagcucaguguuccucucagauc
hsa-miR-3146	caugcuaggacagaaagaaugg	cuucuuucuauccuaguauaacu	cuucuuucuauccuaguauaacuugaagaauucaaauagucaugcuaggacagaaagaaugg
hsa-miR-655-3p	auaauacaugguuaaccucuuu	agagguuauccguguuauguu	agagguuauccguguuauguucgcuucauucaucaugaauaauacaugguuaaccucuuu
hsa-miR-32-5p	uauugcacauuacuaaguugca	caauuuagugugugugauauu	uauugcacauuacuaaguugcauguugucacggccucaaugcaauuuagugugugugauauu
pha-miR-30e	uguaaacaauuccuaggcaauau	auuccuagaaauuguucauaau	uguaaacaauuccuaggcaauauguauaauguuuauaagacauuccuagaaauuguucauaau

Id	Mature sequence	Star sequence	Consensus precursor sequence
pbv-mir-153-2	guuaaucauuggucucuguguccu	gauggagacuaaugauuaauguc	guuaaucauuggucucuguguccuuuuucuagcccaggauggagacuaaugauuaauguc
hsa-mir-548x	guaaaaacugcaaucacuuuca	aaaaguaauugcgguuuugggc	aaaaguaauugcgguuuugggcuuuacuuuuaaaugguaaaaacugcaaucacuuuca
mml-miR-139-5p	ucuacagugcacgugucuccagu	uggagacgcggcccuguuggagu	ucuacagugcacgugucuccaguguggcuccgaggcuggagacgcggcccuguuggagu
hsa-miR-127-3p	ucggauccgucugagcuuggcu	cugaagcucagagggcucugauu	cugaagcucagagggcucugauucagaaagaucaucggauccgucugagcuuggcu
mml-mir-130a	cagugcaauguuaaaagggcauu	gcucuuuucacauugugcuacu	gcucuuuucacauugugcuacugucugcaccugucacuagcagugcaauguuaaaagggcauu
mml-mir-128a	ucacagugaaccggucucuuu	cggggccguagcacugucugaga	cggggccguagcacugucugagagguuuacauuucucacagugaaccggucucuuu
hsa-miR-362-5p	aauccuuggaaccuaggugugagu	aacacaccuauucaaggauuca	aauccuuggaaccuaggugugagugcuauuucagugcaacacaccuauucaaggauuca
mml-miR-149-5p	ucuggcuccgugucuucacucc	gagggagggacgggggcugugc	ucuggcuccgugucuucacucccguguuuguccgaggagggagggagggacgggggcugugc
hsa-miR-323a-3p	cacauuacacggucgaccucu	aggugguccguggcgcguucgc	aggugguccguggcgcguucgcuuuauuuauggcgcacauuacacggucgaccucu
cja-mir-9959-isomiR	accugcucagcgccacugucc	gacgguggggcuggccagcucu	accugcucagcgccacuguccagggaugugagugcaagacgguggggcuggccagcucu
hsa-miR-892c-3p	cacuguuuccuuucugagugga	uauucaggaaggugccaguca	uauucaggaaggugccagucacuuagauuacaugucacuguuuccuuucugagugga
hsa-miR-193a-5p	ugggucuuugcgggcgagauga	aacuggccuacaaagucccagu	ugggucuuugcgggcgagaugagggugucggaucaacuggccuacaaagucccagu
hsa-miR-99b-5p	aacccguagauccgaucuugu	caagcucgcuucuaugggucugu	aacccguagauccgaucuuguggugaaguggaccgcacaagcucgcuucuaugggucugu
hsa-miR-185-5p	uggagagaaaggcaguuccuga	aggggcuggcuuuccucuggu	uggagagaaaggcaguuccugaugguccccuccucaggggcuggcuuuccucuggu
hsa-miR-320a-3p	uucccuuugucauccuaugccu	gcugggaaggcaaagggacgu	uucccuuugucauccuaugccugagaauauaugaaggaggcugggaaggcaaagggacgu
mml-mir-320b	aaaagcuggguugagagggcga	gccuucucuucccgguucuucc	gccuucucuucccgguucuucccggagucgggaaaagcuggguugagagggcga
hsa-miR-376c-3p	aacauagaggaaauuccacgu	guggauauuccuucuauguuua	guggauauuccuucuauguuuauguuauuuaugguuaaacauagaggaaauuccacgu
mml-let-7a-5p	ugagguaguagguuguauaguu	cuguacagccuccuagcuuucc	ugagguaguagguuguauaguuuagaauuacaucaagggagauaacuguacagccuccuagcuuucc
mml-let-7a-5p	ugagguaguagguuguauaguu	cuauacaaucuacugucuuucc	ugagguaguagguuguauaguuuggggcucugcccugcuaugggauaacuauacaaucuacugucuuucc
mmu-miR-193b-3p	aacuggcccucaaagucccgcu	cgggguuuugagggcgagaug	cgggguuuugagggcgagaugaguuuauguuuuauccaacuggcccucaaagucccgcu
hsa-miR-424-5p	ugagguaguagguuguauaguu	cuauacaaucuacugucuuucc	ugagguaguagguuguauaguuuuagggucacacccaccacugggagauaacuauacaaucuacugucuuucc
mml-mir-15b	cagcagcaauucauguuuugaa	caaaacgugaggcgcugcuaua	cagcagcaauucauguuuugaaguguucuaaaugguucaaaacgugaggcgcugcuaua
hsa-miR-212-5p	accuuggcucuagacugcuuacu	uaacagucuccagucacggcc	accuuggcucuagacugcuuacugcccgggccgcccucaguaacagucuccagucacggcc
hsa-mir-101-1	uacaguacugugauaacugaag	caguuaucacagugcugaugc	caguuaucacagugcugaugcuguccauucuaaagguacaguacugugauaacugaag

TABLE II

Differentially regulated miRNAs after 24 h of infection in Vero cells

Like seed miRNAs	miRDeep2.score	log2FoldChange	Regulation in response of infection
hsa-mir-31	1033.1	9.1	Upregulation
mml-mir-203	5.5	9.0	Upregulation
mmu-miR-214-3p	1231.2	8.7	Upregulation
mml-let-7a-5p	85755	8.0	Upregulation
mml-mir-199a-1	40792.6	7.6	Upregulation
pha-miR-30e	83.2	7.5	Upregulation
mml-let-7a-5p	27520.7	6.7	Upregulation
sfr-mir-10499b-1	35.1	6.4	Upregulation
mml-mir-199a-2	26777.6	6.2	Upregulation
mmu-miR-214-3p	5	5.9	Upregulation
hsa-miR-146a-5p	22300.5	4.9	Upregulation
mml-mir-15b	481.5	4.9	Upregulation
mmu-miR-7b-5p	4.9	4.7	Upregulation
mmu-miR-7b-5p	320841	4.7	Upregulation
mml-mir-125b-1	68118.4	4.6	Upregulation
mml-mir-125b-1	85764	4.5	Upregulation
hsa-miR-652-3p	5036.9	4.4	Upregulation
hsa-miR-219a-1-3p	350.5	4.4	Upregulation
mml-let-7a-5p	1259279.4	4.2	Upregulation
mml-mir-15b	1812.1	4.2	Upregulation
oga-miR-505	1499.4	4.1	Upregulation
mml-miR-149-5p	8950.2	3.9	Upregulation
hsa-miR-99b-5p	459183.7	3.9	Upregulation
hsa-miR-99b-5p	9183.5	3.8	Upregulation
mmu-miR-200a-3p	5.3	3.7	Upregulation
hsa-miR-196a-5p	7870.3	3.7	Upregulation
hsa-miR-92b-3p	252	3.6	Upregulation
mml-mir-1-1	4.9	3.6	Upregulation
mml-mir-1-1	5.1	3,6	Upregulation
hsa-miR-10b-5p	325617.8	3.5	Upregulation
mml-mir-130a	5.4	3.5	Upregulation
tgu-mir-215	2728.7	3.4	Upregulation
hsa-miR-330-5p	692.8	3.4	Upregulation
mmu-mir-615	1747.9	3.3	Upregulation
mml-mir-15b	219.5	3.3	Upregulation
hsa-miR-450a-5p	2261.3	3.3	Upregulation
hsa-miR-450a-5pIsomiR	2273.2	3.3	Upregulation
ath-MIR854a	41.6	3.2	Upregulation
hsa-miR-301a-5p	453.2	3.2	Upregulation
pha-mir-26b	27632.7	3.1	Upregulation
mmu-mir-542	6901	3.1	Upregulation
mml-mir-15b	989.5	3.1	Upregulation
hsa-miR-656-3p	5046.4	-3.1	Downregulation
mml-mir-548g	50.7	-3.2	Downregulation
mml-miR-890-3p	138.7	-3.3	Downregulation
hsa-mir-892a	175	-4.1	Downregulation
